# Factors associated with a nonresponse to prone positioning in patients with severe acute respiratory distress syndrome due to SARS-CoV-2

**DOI:** 10.5935/2965-2774.20230343-en

**Published:** 2023

**Authors:** Oscar Orlando Sanabria-Rodríguez, Sergio Leonardo Cardozo-Avendaño, Oscar Mauricio Muñoz-Velandia

**Affiliations:** 1 Hospital Universitario San Ignacio, Pontificia Universidad Javeriana - Bogotá, Colombia; 2 Pontificia Universidad Javeriana - Bogotá, Colombia

**Keywords:** Respiratory distress syndrome, Respiration, artificial, Intubation, intratracheal, Prone position, Hypoxia, SARS-CoV-2, Coronavirus infections, COVID-19

## Abstract

**Objective:**

To identify risk factors for nonresponse to prone positioning in mechanically ventilated patients with COVID-19-associated severe acute respiratory distress syndrome and refractory hypoxemia in a tertiary care hospital in Colombia.

**Methods:**

Observational study based on a retrospective cohort of mechanically ventilated patients with severe acute respiratory distress syndrome due to SARS-CoV-2 who underwent prone positioning due to refractory hypoxemia. The study considered an improvement ≥ 20% in the PaO_2_/FiO_2_ ratio after the first cycle of 16 hours in the prone position to be a ‘response’. Nonresponding patients were considered cases, and responding patients were controls. We controlled for clinical, laboratory, and radiological variables.

**Results:**

A total of 724 patients were included (58.67 ± 12.37 years, 67.7% males). Of those, 21.9% were nonresponders. Mortality was 54.1% for nonresponders and 31.3% for responders (p < 0.001). Variables associated with nonresponse were time from the start of mechanical ventilation to pronation (OR 1.23; 95%CI 1.10 - 1.41); preintubation PaO_2_/FiO_2_ ratio (OR 0.62; 95%CI 0.40 - 0.96); preprone PaO_2_/FiO_2_ ratio (OR 1.88. 95%CI 1.22 - 2.94); and radiologic multilobe consolidation (OR 2.12; 95%CI 1.33 - 3.33) or mixed pattern (OR 1.72; 95%CI 1.07 - 2.85) compared with a ground-glass pattern.

**Conclusion:**

This study identified factors associated with nonresponse to prone positioning in patients with refractory hypoxemia and acute respiratory distress syndrome due to SARS-CoV-2 receiving mechanical ventilation. Recognizing such factors helps identify candidates for other rescue strategies, including more extensive prone positioning or extracorporeal membrane oxygenation. Further studies are needed to assess the consistency of these findings in populations with acute respiratory distress syndrome of other etiologies.

## INTRODUCTION

Since the initial appearance of severe acute respiratory syndrome coronavirus 2 (SARS-CoV-2) in Wuhan (China), more than 600 million infections have been reported worldwide. The virus killed more than 6.4 million people through September 2022 and cost more than 41 billion euros.

Coronavirus disease 2019 (COVID-19) has a spectrum of manifestations, from an asymptomatic form to a multisystemic illness.^(1)^ Lung involvement is the best-understood presentation and may cause acute respiratory distress syndrome (ARDS).^(2)^ Without adequate treatment, this condition may lead to death. Management of ARDS includes protective mechanical ventilation (MV) with low tidal volumes, optimal positive end-expiratory pressure (PEEP),^(3,4)^ and dexamethasone.^(5)^ Despite those interventions, some patients present refractory hypoxemia and require the use of neuromuscular blocking agents^(6)^ and prone positioning.^(7,8)^ The prone-positioning strategy aims to improve oxygenation by recruiting alveolar capillary units in dependent posterior regions, establishing alveolar homogeneity, reducing the heart weight over the lower left lobe of the lung, minimizing the influence of abdominal pressure, improving the ventilation/perfusion (V/Q) ratio, reducing stress, and providing more homogeneous lung distension.^(9)^ These mechanisms have reduced the mortality rate of COVID-19-associated ARDS.^(10)^ A percentage of nonresponding patients, however, require late use of other rescue strategies, which reduces the clinical recovery and survival rate.

Factors related to nonresponse to prone positioning remain unclear. Observational studies suggest that such factors include time between the onset of clinical disease and start of pronation, some radiological patterns, hypoxia severity, intrapulmonary shunt, and obesity.^(11)^

This study aimed to identify risk factors for nonresponse to prone positioning in mechanically ventilated patients with COVID-19-associated severe ARDS and refractory hypoxemia in a tertiary care hospital in Colombia.

## METHODS

This was an observational study based on a retrospective cohort of patients older than 18 years suffering from severe ARDS associated with SARS-CoV-2. Subjects were admitted from June 2020 to February 2022 into the intensive care unit (ICU) of *Hospital Universitario San Ignacio* (HUSI) in Bogotá, Colombia. Diagnosis of severe ARDS was based on Berlin criteria,^(12)^ and SARS-CoV-2 infection was tested by polymerase chain reaction (PCR) or antigen test. The cohort included patients who required MV and, due to refractory hypoxemia (partial pressure of oxygen/fraction of inspired oxygen - PaO_2_/FiO_2_ persistently < 150), underwent prone positioning. The study excluded patients with interrupted prone positioning due to reasons other than nonresponse and patients whose MV started at another institution. The institutional committee of ethics approved the study (Act No. MI 015-2022), which was performed in accordance with the ethical standards laid down in the 1964 Declaration of Helsinki and its later amendments.

In the study cohort, indications for invasive MV were PaO_2_/FiO_2_ < 150 or clinical signs of respiratory failure. In accordance with previous studies, protective MV parameters included tidal volume (Vt) in 6 - 8mL/kg; plateau airway pressure (Ppl) < 25cmH_2_O; driving pressure (DP) < 15cmH_2_O; and PEEP set by individual pulmonary mechanics. Immediately after the start of MV, an arterial blood gas test was performed. Qualifying patients with refractory hypoxemia (PaO_2_/FiO_2_ < 150) received neuromuscular blockage for 48 hours and were placed in the prone position for 16 hours followed by a subsequent change to the supine position for 8 hours. Arterial blood gas check-ups assessed responses at hour 15 of prone positioning and at hour 7 after the change back to the supine position. The study considered an improvement ≥ 20% in the PaO_2_/FiO_2_ ratio after the first cycle of pronation as a ‘response’. Nonresponding patients were considered cases, and responding patients were controls using criteria based on previous observational trials.^(13)^

With a standardized instrument, the authors collected data from electronic clinical records. The collected data comprised clinical variables, comorbidities, paraclinical test results, severity at admission to the ICU, features of pulmonary mechanics, and use of vasoactive or sedative drugs. Paraclinical variables included complete blood count, serum markers with prognostic value (D-dimer > 1,000ng/mL), and arterial blood gas measurements taken immediately prior to the start of ventilation and pronation cycles. Radiological findings considered three possible patterns, namely ground-glass opacities, lung consolidation, or mixed pattern, each reflecting different stages of the disease. Specialists in radiology used standardized criteria to classify findings in thoracic imaging. Simplified Acute Physiology Score 3 (SAPS 3) was used to evaluate severity at admission to the ICU.

We described categorical variables with absolute and relative frequencies. For continuous variables, we presented mean and standard deviation or median and interquartile range (IQR), according to data distribution. The Shapiro‒Wilk test was used to assess the normality assumption. Comparisons between cases and controls were performed using Student’s t test or Mann‒Whitney U test, chi-square test, or Fisher’s exact test, depending on the variable type.

Kaplan‒Meier curves and log rank tests were used to compare survival functions between responders and nonresponders to pronation. Assessment of explanatory variables used recategorization for body mass index, preintubation and prone PaO_2_/FiO_2_ ratios, prone pH, static compliance, Ppl, DP, and PEEP. A multivariate logistic regression model then assessed the strength of association among selected variables using backward stepwise variable elimination. A p value < 0.05 was considered statistically significant. Statistical analysis was performed using IBM software Statistical Package for the Social Sciences (SPSS), version 25.0.

## RESULTS

The study included 724 patients with COVID-19 and severe ARDS who received MV and who, due to refractory hypoxemia, underwent prone positioning. The patients’ mean age was 58.67 ± 12.37 years, and the majority were males (67.68%). One hundred fifty-nine patients (21.9%) did not respond to pronation. The median PaO_2_/FiO_2_ variation was 62.8% in responders (IQR 42.85 - 100) and 2.7% (IQR 7.63 - 11.36) in nonresponders.

**Figure 1:**
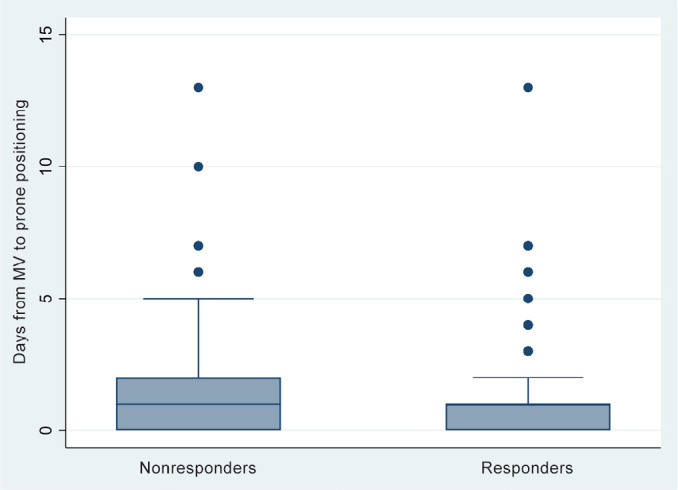
Days from the start of mechanical ventilation to prone positioning for nonresponders and responders.

Table 1 shows patient characteristics by response to prone positioning. Nonresponders had higher D-dimer levels and prepronation PaO_2_/FiO_2_ ratios, more frequent lung consolidation, more frequent need for 3 or more sedatives, and a longer time between the start of MV and the start of pronation (Figure 1). Nonresponders also had higher mortality rates (54.1% *versus* 31.3%; p < 0.001). Kaplan‒Meier curves (Figure 1S - Supplementary material) and log rank tests (p < 0.001) demonstrated worse survival function for nonresponders.

**Table 1:** Patient characteristics and response to prone positioning

Variable	Total n = 724	No response to pronation n = 159	Response to pronation n = 565	p value
Age (years)	58.67 ± 12.37	58.59 ± 12.68)	58.69 ± 12.29	0.927
Sex, male	490 (67.68)	112 (70.44)	378 (66.90)	0.399
Symptom (days)	7.22 ± 4.48	6.86 ± 3.93	7.32 ± 4.62	0.398
BMI (kg/m^2^)	29.19 ± 5.49	28.79 ± 5.49	29.30 ± 5.49	0.298
Overweight	289 (39.92)	66 (41.51)	223 (39.47)	0.700
Obesity I	182 (25.14)	38 (23.90)	144 (25.49)	
Obesity II	61 (8.43)	10 (6.29)	51 (9.03)	
Obesity III	28 (3.87)	5 (3.14)	23 (4.07)	
COPD	39 (5.40)	6 (3.80)	33 (5.85)	0.313
Number of comorbidities	1.46 (1.37)	1.46 (1.33)	1.46 (1.30)	0.883
Basal glomerular filtration rate (mL/min/m^2^)	104.86 ± 51.04	101.74 ± 56.54	105.74 ± 49.39	0.397
Hemoglobin (g/dL)	13.44 ± 1.95	13.11 ± 2.20)	13.53 ± 1.87	0.018
D-dimer (ng/mL)	1,646.95 ± 1867.31	1,870.33 ± 2064.97	1,585.11 ± 1805.93	0.022
Creatinin (mg/dL)	1.06 ± 0.90	1.18 ± 1.11	1.03 ± 0.82	0.285
SAPS 3 score at ICU admission	64 (61 - 70)	64 (62 - 71)	63 (60 - 69.5)	0.207
X-ray pattern				
Ground-glass	282 (38.95)	43 (27.04)	239 (42.30)	0.002
Consolidation	236 (32.60)	65 (40.88)	171 (30.27)	
Mixed	206 (28.45)	51 (33.08)	155 (27.43)	
Preintubation PaO_2_/FiO_2_	101.03 ± 35.54	102.13 ± 37.28	100.71 ± 35.06	0.655
Prepronation PaO_2_/FiO_2_	109.46 ± 32.73	124.74 ± 35.89	105.16 ±3 0.47	< 0.001
PaO_2_/FiO_2_ ratio change (%)	50.82 (24.25 - 87.09)	2.70 (-7.63 - 11.36)	62.88 (42.85 - 100)	< 0.001
Compliance (mL/cmH_2_O)	37.58 (12.07)	38.57 (13.09)	37.30 (11.74)	0.239
Plateau pressure (cmH_2_O)	22.60 (2.92)	22.68 (2.93)	22.58 (2.92)	0.709
PEEP (cmH_2_O)	11.50 (1.62)	11.28 (1.67)	11.56 (1.62)	0.054
Driving pressure (cmH_2_O)	11.09 (2.69)	11.40 (2.88)	11.01 (2.63)	0.100
Use of vasoactives				
0	375 (51.87)	80 (50.31)	295 (52.30)	0.230
1	334 (46.06)	73 (45.91)	261 (46.10)	
2	15 (2.07)	6 (3.77)	9 (1.60)	
Use of sedatives				
0 - 1	5 (0.69)	1 (0.63)	4 (0.67)	< 0.001
2	619 (85.48)	123 (77.36)	496 (87.77)	
3 - 4	100 (13.83)	35 (22.01)	65 (11.53)	
Dexamethasone	707 (97.65)	155 (97.48)	552 (97.70)	0.775
Days on MV	9.61 ± 5.19	9.38 ± 5.09	9.68 ± 5.22	0.533
Days in ICU	15.38 ± 10.15	14.55 ± 10.44	15.61 ± 10.07	0.877
Days on MV and pronation	1 (0 - 1)	1 (0 - 2)	1 (0 - 1)	< 0.001
Deaths	263 (36.33)	86 (54.09)	177 (31 . 33)	< 0.001

BMI - body-mass index; COPD - chronic obstructive pulmonary disease; SAPS 3 - Simplified Acute Physiology Score 3; ICU - intensive care unit; PaO_2_/FiO_2_ - partial pressure of oxygen/fraction of inspired oxygen; PEEP - positive end-expiratory pressure; MV - mechanical ventilation. Results expressed as mean ± standard deviation, n (%) or median (interquartile range).

MV - mechanical ventilation.

Table 2 presents the PaO_2_/FiO_2_ ratio change rate by basal arterial blood gas measurement, radiographic pattern, and ventilatory strategy. The PaO_2_/FiO_2_ change was lower when preintubation or prepronation PaO_2_/FiO_2_ was higher, when DP was ≥ 15, and in patients who received more sedatives.

**Table 2:** Partial pressure of oxygen/fraction of inspired oxygen ratio change by radiological pattern, basal arterial gas measurement, and ventilatory parameters

Variable	% PaO_2_/FiO_2_ change	p value
	Median (IQR)	
Radiological pattern		0.049
Ground-glass opacities	56.65 (30.15 - 85.71)	
Consolidation	47.14 (14.49 - 86.17)	
Mixed	47.75 (19.23 - 88.99)	
Preintubation PaO_2_/FiO_2_		0.011
< 100	55.55 (25.37 - 92)	
100 - < 150	50 (25.92 - 87.61)	
≥ 150	37.32 (10.29 - 62.68)	
Prepronation PaO_2_/FiO_2_		< 0.001
<100	72.29 (35.20 - 127.38)	
100 - < 150	46.97 (23.09 - 70.20)	
≥ 150	17.83 (2.50 - 42.22)	
pH		0.902
< 7.3	50.60 (24.65 - 82.22)	
7.3 - < 7.4	53.29 (20.59 - 88.15)	
≥ 7.4	48.23 (25.95 - 92.30)	
Compliance (mL/cmH_2_O)		0.252
< 20	64.53 (39.05 - 90.18)	
20 - < 30	46.92 (21.00 - 86.30)	
30 - < 40	52.93 (25.95 - 88.23)	
40 - < 50	50 (19.59 - 80)	
≥ 50	57.20 (19.80 - 94.65)	
Plateau pressure		0.867
< 20	59.29 (28.90 - 87.09)	
20 - < 25	50.53 (24.60 - 83.57)	
25 - <3 0	49.41 (20.00 - 93.10)	
≥ 30	34.59 (20.70 - 139.18)	
Driving pressure		0.042
< 10	56.75 (28.07 - 87.61)	
10 - < 15	50.51 (24.74 - 87.61)	
≥ 15	39.50 (12.83 - 67.24)	
Number of sedatives used		< 0.001
1	116.02 (41.95 - 149.16)	
2	53.73 (26.15 - 92.30)	
3	29.78 (8.6 - 62.90)	
4	10.29 (-5.26 - 17.00)	
Death		< 0.001
No	56.36 (28.24 - 87.61)	
Yes	40.50 (10.52 - 82.92)	

PaO_2_/FiO_2_ - partial pressure of oxygen/fraction of inspired oxygen; IQR - interquartile range.

The logistic regression model showed that the chance of not responding to prone positioning increased significantly each day after the start of MV (Table 3). The model also showed that the likelihood of not responding was higher with a lung consolidation or mixed radiological pattern than with a ground-glass pattern.

**Table 3:** Raw and multivariate models for factors associated with nonresponse to prone positioning

Variable	Raw OR (95%CI)	Adjusted OR	95%CI	p value
Days from mechanical ventilation to pronation	1.26 (1.14 - 1.42)	1.23	1.10 - 1.41	< 0.001
Radiological pattern				
Ground-glass	Ref	Ref		
Consolidation	2.12 (1.39 - 3.33)	2.12	1.33 - 3.33	0.002
Mixed	1.85 (1.16 - 2.94)	1.72	1.07 - 2.85	0.026
Preintubation PaO_2_/FiO_2_				
< 100	Ref	Ref		
100 - < 150	0.79 (0.52 - 1.17)	0.62	0.40 - 0.96	0.030
≥ 150	1.53 (0.83 - 2.70)	0.95	0.51 - 1.78	0.861
Prepronation PaO_2_/FiO_2_				
< 100	Ref	Ref		
100 - < 150	1.66 (1.10 - 2.56)	1.88	1.22 - 2.94	0.005
≥ 150	7.14 (4.00 - 12.50)	7.14	4.00 - 12.50	< 0.001

OR - odds ratio; 95%CI - 95% confidence interval; PaO_2_/FiO_2_ - partial pressure of oxygen/fraction of inspired oxygen. Hosmer-Lemeshow test: p value = 0.493.

**Figure 2:**
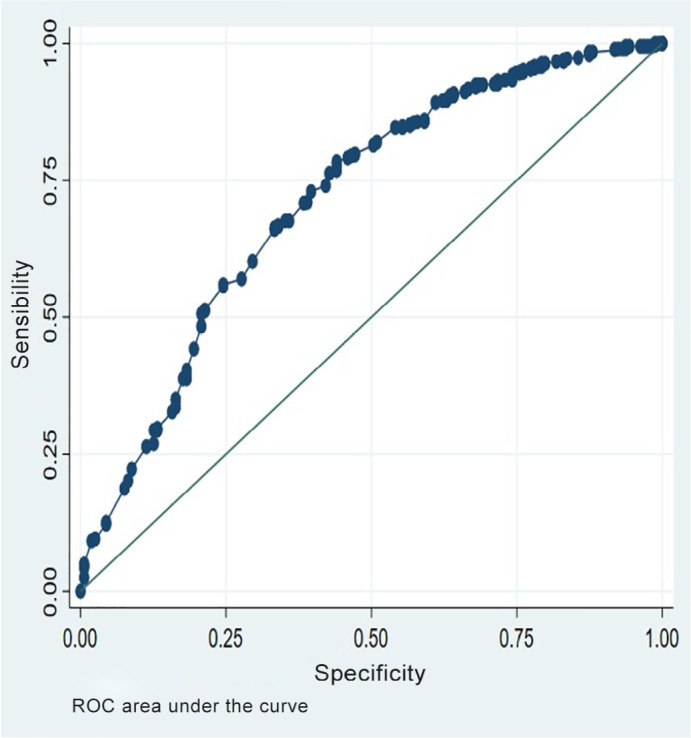
ROC curve assessing the model’s discrimination capacity for predicting lack of response to prone positioning.

We found a low correlation between preintubation PaO_2_/FiO_2_ and prepronation PaO_2_/FiO_2_ (Spearman correlation test 0.37) (Figure 2S - Supplementary material), so both variables were evaluated in the model. The likelihood of not responding to prone positioning was lower in patients with a preintubation PaO_2_/FiO_2_ of 100 - 150 than in patients with a preintubation PaO_2_/FiO_2_ < 100. In contrast, patients with preprone PaO_2_/FiO_2_ of 100 - 150 were twice as likely to not respond to the prone position as patients with PaO_2_/FiO_2_ < 100. That probability was even higher for patients with PaO_2_/FiO_2_ >150 (Table 3).

Assessment of discrimination capacity showed that the model correctly predicted nonresponse to prone positioning in 79.28% of cases, with proper discrimination capacity (area under the curve - AUC 0.713) (Figure 2).

ROC - receiver operating characteristic.

## DISCUSSION

This study documented a response in 78% of patients in prone positioning, similar to the 70% success rate reported in the literature.^(14)^ Factors associated with nonresponse are radiological pattern, time from VM to prone position, and PaO_2_/FiO_2_ before intubation and pronation.

Multiple authors have attempted to find scores and patterns^(11)^ predicting response to prone positioning^(15,16)^ but have found no clear results. This study showed that the time from the start of MV to prone positioning is a relevant factor for response. This is consistent with findings by Guerín’s reviews of clinical trials.^(17)^ Clearly, delaying the start of lung protective measurements favors inflammation and patient self-induced lung injury (P-SILI).^(18)^

The chest radiological pattern was another variable associated with response. Mixed pattern and consolidation were associated with nonresponse. This study considered three radiological patterns based on COVID-19 inflammatory physiology. Each pattern describes a different stage of the disease, revealing extension and severity of parenchymatous involvement. Bedside ultrasound may also play an important role in predicting response.^(19-21)^

Finally, preintubation and preprone PaO_2_/FiO_2_ were inversely associated with response. The lower the preprone PaO_2_/FiO_2_ was, the higher the response likelihood. Interpretation of these findings, however, must be made cautiously. Mathematically, any change in response taken from lower values will have a higher percentage of variation compared to a slightly higher basal value, even when the absolute change is similar. Similar responses have been evident since the first studies in the literature by Blanch et al.^(22)^ Another possible explanation is that prone PaO_2_/FiO_2_ ratio deterioration during the ICU stay could be a potential tool to predict prone response. Future studies will be necessary to evaluate this hypothesis.

Although it was not a goal of this study, we revealed significant mortality differences between nonresponding and responding patients (56.36 *versus* 40.5%, p ≤ 0.001) and significant survival functions. These results vary from results reported by van Meenen et al., which showed no differences between the two groups.^(13)^

Considering that the inflammatory physiology in ARDS affects not only the lung but also endothelial structures, other variables may be relevant in response to prone positioning. These include D-dimer, hemoglobin indicating anemia, Ppl, and DP. Although the multivariate analysis revealed no strong association between those variables and response, they should be taken into account.

The number of sedatives is another variable to mention. The univariate analysis showed a significantly lower response rate in patients requiring a higher number of sedatives. Larger parenchymatous involvement may require increased respiratory control, with a greater need for sedation.

A strength of this study is the number of patients included. This is the largest cohort of ADRS patients with prone positioning to date. Additionally, the study´s 1:3 case:control ratio allows better precision in the assessment of the strength of association. Systematic selection of ventilatory parameters through pulmonary mechanics, systematic selection of initial prone positioning time (16 hours), and generalized use of neuromuscular blockage for the first 48 hours were based on classical studies, such as PROSEVA.^(7)^ These characteristics assured homogeneity in the ventilation method.

Limitations of this study include its retrospective, observational, unicentric nature. In addition, there was a lack of relevant data, such as Troponin levels, a possible variable of association. Assessment of the outcome of interest, however, was not compromised thanks to systematic data collection from the hospital MV protocol. Finally, it is an open question whether oxygenation improvement should be the ultimate goal. Should clinicians be permissive with the use of different protection strategies (including prone positioning) for patients with hypoxemia^(23)^ to avoid worsening inflammation until the ARDS-triggering noxious stimulus resolves?

The results in this study suggest that it is possible to establish response-predicting scores. These findings support the early use of other rescue strategies, including more extensive prone positioning or extracorporeal membrane oxygenation in patients with ARDS of any etiology,^(24)^ to manage refractory hypoxemia.

## CONCLUSION

Some factors are probably associated with a nonresponse to prone positioning in patients with SARS-CoV-2-associated severe acute respiratory distress syndrome with mechanical ventilation and refractory hypoxemia. These include the time from the start of mechanical ventilation until prone positioning, the preprone partial pressure of oxygen/fraction of inspired oxygen value, and a mixed or multilobar-consolidation radiological pattern.

Prospective studies are required to assess a possible association among nonresponse and other relevant variables, such as Sequential Organ Failure Assessment score, acute lung thromboembolism, and myocardiopathy. Additionally, new studies are required to determine whether the findings in this study are consistent in populations with acute respiratory distress syndrome from causes other than COVID-19.

## References

[1] Seyed Hosseini E, Riahi Kashani N, Nikzad H, Azadbakht J, Hassani Bafrani H, Haddad Kashani H. (2020). The novel coronavirus Disease-2019 (COVID-19):mechanism of
action, detection and recent therapeutic strategies. Virology.

[2] Lentz S, Roginski MA, Montrief T, Ramzy M, Gottlieb M, Long B. (2020). Initial emergency department mechanical ventilation strategies
for COVID-19 hypoxemic respiratory failure and ARDS. Am J Emerg Med.

[3] Amato MB, Meade MO, Slutsky AS, Brochard L, Costa EL, Schoenfeld DA (2015). Driving pressure and survival in the acute respiratory distress
syndrome. N Engl J Med.

[4] Brower RG, Lanken PN, MacIntyre N, Matthay MA, Morris A, Ancukiewicz M, Schoenfeld D, Thompson BT, National Heart, Lung, and Blood Institute ARDS Clinical Trials
Network (2004). Higher versus lower positive end-expiratory pressures in patients
with the acute respiratory distress syndrome. N Engl J Med.

[5] Horby P, Lim WS, Emberson JR, Mafham M, Bell JL, Linsell L, RECOVERY Collaborative Group (2021). Dexamethasone in hospitalized patients with
COVID-19. N Engl J Med.

[6] Papazian L, Forel JM, Gacouin A, Penot-Ragon C, Perrin G, Loundou A, Jaber S, Arnal JM, Perez D, Seghboyan JM, Constantin JM, Courant P, Lefrant JY, Guérin C, Prat G, Morange S, Roch A, ACURASYS Study Investigators (2010). Neuromuscular blockers in early acute respiratory distress
syndrome. N Engl J Med.

[7] Guérin C, Reignier J, Richard JC, Beuret P, Gacouin A, Boulain T, Mercier E, Badet M, Mercat A, Baudin O, Clavel M, Chatellier D, Jaber S, Rosselli S, Mancebo J, Sirodot M, Hilbert G, Bengler C, Richecoeur J, Gainnier M, Bayle F, Bourdin G, Leray V, Girard R, Baboi L, Ayzac L, PROSEVA Study Group (2013). Prone positioning in severe acute respiratory distress
syndrome. N Engl J Med.

[8] Marik PE, Iglesias J. A (1997). “prone dependent” patient with severe adult respiratory distress
syndrome. Crit Care Med.

[9] Lee HY, Cho J, Kwak N, Choi SM, Lee J, Park YS (2020). Improved oxygenation after prone positioning may be a predictor
of survival in patients with acute respiratory distress
syndrome. Crit Care Med.

[10] Thompson BT. (2013). Prone positioning for 16 h/d reduced mortality more than supine
positioning in early severe ARDS. Ann Intern Med.

[11] Koulouras V, Papathanakos G, Papathanasiou A, Nakos G. (2016). Efficacy of prone position in acute respiratory distress syndrome
patients: a pathophysiology-based review. World J Crit Care Med.

[12] Ranieri VM, Rubenfeld GD, Thompson BT, Ferguson ND, Caldwell E, Fan E, ARDS Definition Task Force (2012). Acute respiratory distress syndrome: the Berlin
Definition. JAMA.

[13] van Meenen DM, Roozeman JP, Serpa Neto A, Pelosi P, Gama de Abreu M, Horn J, Cremer OL, Paulus F, Schultz MJ, MARS Consortium (2019). Associations between changes in oxygenation, dead space and
driving pressure induced by the first prone position session and mortality
in patients with acute respiratory distress syndrome. J Thorac Dis.

[14] Kallet RH. (2015). A comprehensive review of prone position in ARDS. Respir Care.

[15] Chen YY, Kuo JS, Ruan SY, Chien YC, Ku SC, Yu CJ (2022). Prognostic value of computed tomographic findings in acute
respiratory distress syndrome and the response to prone
positioning. BMC Pulm Med.

[16] Papazian L, Paladini MH, Bregeon F, Thirion X, Durieux O, Gainnier M (2002). Can the tomographic aspect characteristics of patients presenting
with acute respiratory distress syndrome predict improvement in
oxygenation-related response to the prone position?. Anesthesiology.

[17] Guérin C, Albert RK, Beitler J, Gattinoni L, Jaber S, Marini JJ (2020). Prone position in ARDS patients: why, when, how and for
whom. Intensive Care Med.

[18] Cruces P, Retamal J, Hurtado DE, Erranz B, Iturrieta P, González C (2020). A physiological approach to understand the role of respiratory
effort in the progression of lung injury in SARS-CoV-2
infection. Crit Care.

[19] Haddam M, Zieleskiewicz L, Perbet S, Baldovini A, Guervilly C, Arbelot C, Noel A, Vigne C, Hammad E, Antonini F, Lehingue S, Peytel E, Lu Q, Bouhemad B, Golmard JL, Langeron O, Martin C, Muller L, Rouby JJ, Constantin JM, Papazian L, Leone M, CAR’Echo Collaborative Network; AzuRea Collaborative
Network (2016). Lung ultrasonography for assessment of oxygenation response to
prone position ventilation in ARDS. Intensive Care Med.

[20] Guerin C, Gattinoni L. (2016). Assessment of oxygenation response to prone position ventilation
in ARDS by lung ultrasonography. Intensive Care Med.

[21] Prat G, Guinard S, Bizien N, Nowak E, Tonnelier JM, Alavi Z (2016). Can lung ultrasonography predict prone positioning response in
acute respiratory distress syndrome patients?. J Crit Care.

[22] Blanch L, Mancebo J, Perez M, Martinez M, Mas A, Betbese AJ (1997). Short-term effects of prone position in critically ill patients
with acute respiratory distress syndrome. Intensive Care Med.

[23] Abdelsalam M, Cheifetz IM. (2010). Goal-directed therapy for severely hypoxic patients with acute
respiratory distress syndrome: permissive hypoxemia. Respir Care.

[24] Chiumello D, Brioni M. (2016). Severe hypoxemia: which strategy to choose. Crit Care.

